# Spermidine promotes *Bacillus subtilis* biofilm formation by activating expression of the matrix regulator *slrR*

**DOI:** 10.1074/jbc.M117.789644

**Published:** 2017-05-25

**Authors:** Laura Hobley, Bin Li, Jennifer L. Wood, Sok Ho Kim, Jacinth Naidoo, Ana Sofia Ferreira, Maxim Khomutov, Alexey Khomutov, Nicola R. Stanley-Wall, Anthony J. Michael

**Affiliations:** From the Departments of ‡ Pharmacology and; ¶ Biochemistry, University of Texas Southwestern Medical Center at Dallas, Dallas, Texas 75390,; the § Division of Molecular Microbiology, School of Life Sciences, University of Dundee, Dundee DD15EH, Scotland, United Kingdom, and; the ‖ Engelhardt Institute of Molecular Biology, Russian Academy of Sciences, Vavilov Street 32, Moscow 119991, Russia

**Keywords:** bacteria, biofilm, polyamine, spermidine, transcriptomics, Bacillus subtilis, agmatine, aminopropyl, exopolysaccharide, slrR

## Abstract

Ubiquitous polyamine spermidine is not required for normal planktonic growth of *Bacillus subtilis* but is essential for robust biofilm formation. However, the structural features of spermidine required for *B. subtilis* biofilm formation are unknown and so are the molecular mechanisms of spermidine-stimulated biofilm development. We report here that in a spermidine-deficient *B. subtilis* mutant, the structural analogue norspermidine, but not homospermidine, restored biofilm formation. Intracellular biosynthesis of another spermidine analogue, aminopropylcadaverine, from exogenously supplied homoagmatine also restored biofilm formation. The differential ability of C-methylated spermidine analogues to functionally replace spermidine in biofilm formation indicated that the aminopropyl moiety of spermidine is more sensitive to *C*-methylation, which it is essential for biofilm formation, but that the length and symmetry of the molecule is not critical. Transcriptomic analysis of a spermidine-depleted *B. subtilis speD* mutant uncovered a nitrogen-, methionine-, and *S*-adenosylmethionine-sufficiency response, resulting in repression of gene expression related to purine catabolism, methionine and *S*-adenosylmethionine biosynthesis and methionine salvage, and signs of altered membrane status. Consistent with the spermidine requirement in biofilm formation, single-cell analysis of this mutant indicated reduced expression of the operons for production of the exopolysaccharide and TasA protein biofilm matrix components and SinR antagonist *slrR*. Deletion of *sinR* or ectopic expression of *slrR* in the spermidine-deficient Δ*speD* background restored biofilm formation, indicating that spermidine is required for expression of the biofilm regulator *slrR*. Our results indicate that spermidine functions in biofilm development by activating transcription of the biofilm matrix exopolysaccharide and TasA operons through the regulator *slrR*.

## Introduction

**Figure 1.**
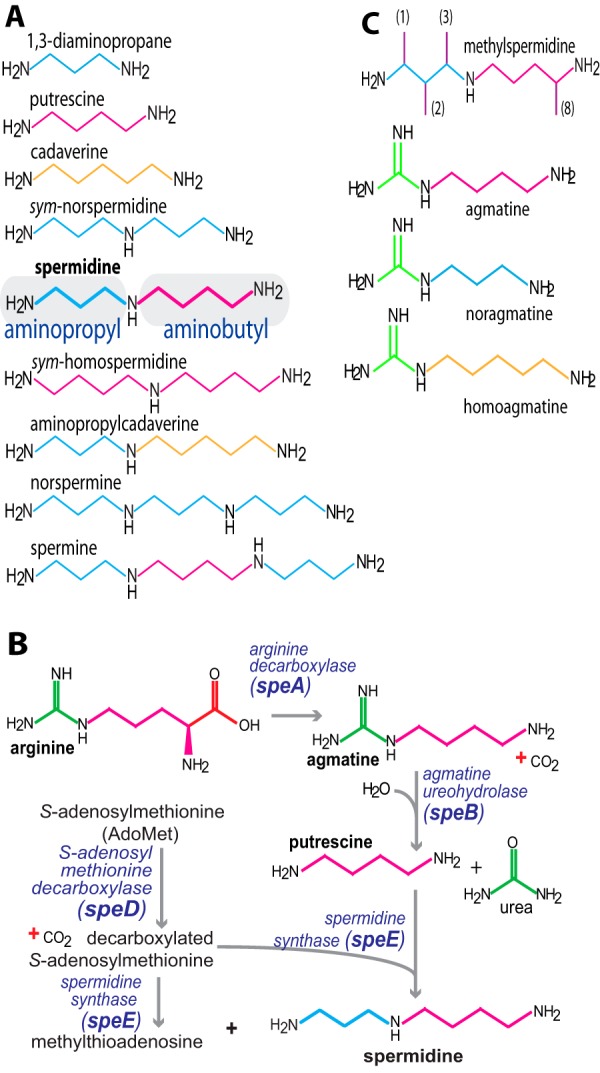
**Polyamine structures and biosynthesis in *B. subtilis*.** *A*, relevant polyamine structures. The first methylene carbon of spermidine is by convention designated as the *1* position and is on the aminopropyl side of the molecule. *B*, pathway for spermidine biosynthesis in *B. subtilis. C*, synthetic polyamine analogues. In the agmatine analogues, the number of methylene carbons in the potential diamine chain are indicated in *parentheses*. Aminopropyl groups are shown in *blue*, aminobutyl groups in *red*, and aminopentyl groups in *tan*.

Most cells synthesize or take up small linear polycationic molecules known as polyamines that are derived from amino acids and are fully protonated at physiological pH (1, 2). The most common polyamine is the triamine spermidine (Fig. 1) and this is the only triamine found in most eukaryotes. Some bacteria also synthesize the triamines *sym*-norspermidine or *sym*-homospermidine, being one methylene carbon shorter or longer, respectively, than spermidine (Fig. 1). Whereas spermidine is likely to be essential for growth of all eukaryotes and archaea (2) due to its role in the hypusine and deoxyhypusine modification of the translation elongation factor IF5A (3–5), the analogous bacterial translation factor E-FP uses lysine rather than the aminobutyl group of spermidine for the equivalent modification (6, 7). The function of hypusinated IF5A and lysinylated EF-P is to facilitate translation of mRNAs encoding proteins containing polyproline tracts (8–10). Although most bacteria synthesize or are capable of taking up polyamines, unlike eukaryotes and archaea they lack a conserved essential function for polyamines, and consequently, their role in planktonic growth varies between species. In *Pseudomonas aeruginosa*, *Campylobacter jejuni,* and *Agrobacterium tumefaciens*, spermidine is essential for planktonic growth (11–13). In contrast, polyamine auxotrophic strains of *Escherichia coli* grow for multiple subcultures under aerobic conditions, albeit with a 40% reduction in growth rate, and a similar reduction in growth rate was observed in polyamine auxotrophic strains of *Yersinia pestis*, *Salmonella enterica* serovar Typhimurium, and *Vibrio cholerae* (14–16). Spermidine auxotrophic strains of the Gram-positive species *Bacillus subtilis* and *Streptococcus pneumoniae* exhibit normal planktonic growth in the absence of spermidine (17–19).

Polyamines also affect the formation of biofilms, which are communities of bacteria enveloped in an extracellular matrix of polysaccharide, proteins, and extracellular DNA (20–23). Exogenous norspermidine was shown to stimulate biofilm formation in *V. cholerae* (24), and endogenous synthesis of norspermidine was required for biofilm formation in this species (25); however, exogenous spermidine inhibited biofilm formation. Synthesis of putrescine and most likely spermidine is essential for biofilm formation in *Y. pestis* (16, 26). Endogenous production of polyamines can also be incompatible with robust biofilm formation. Disruption of putrescine biosynthesis in *Shewanella oneidensis* resulted in increased biofilm formation (27), and disruption of spermidine biosynthesis increased biofilm formation in *A. tumefaciens* (28). Exogenous spermidine repressed biofilm formation in the *A. tumefaciens* spermidine-depleted mutant but norspermidine did not, even though norspermidine could replace the essential function of spermidine in planktonic growth.

In *B. subtilis*, although spermidine biosynthesis is not required for normal growth, it is essential for robust biofilm formation (17, 18), and this role can be replaced by norspermidine but not homospermidine. However, some authors have claimed that low levels of exogenous norspermidine inhibit biofilm formation in *B. subtilis* (29). Two important components of the *B. subtilis* biofilm matrix, the protein TapA and fibrous protein TasA (30), and the exopolysaccharide (31) are dependent on the operons *tapA-sipW-tasA* and *epsA-O*, respectively, for their production. Biofilm formation in *B. subtilis* also requires a hydrophobic coat protein BslA, formerly known as YuaB (32–34), which acts in concert with other matrix components to allow biofilm assembly. Transcription of the *tapA-sipW-tasA* and *epsA-O* operons is strictly regulated by parallel pathways that act to counter transcriptional repression mediated by the AbrB and SinR transcriptional regulators (22). These parallel pathways are controlled by the phosphorylated form of the Spo0A transcription factor, which acts as a nexus for integrating environmental signals that trigger biofilm formation (31, 35). Our goals were to determine which structural features of spermidine or its analogues are necessary for *B. subtilis* biofilm formation, to examine whether alternative polyamines can be synthesized using the endogenous spermidine biosynthetic pathway, to identify cellular processes dependent on spermidine biosynthesis, and to elucidate the role of spermidine in biofilm formation.

## Results

### Polyamine structural specificity in biofilm formation

**Figure 2.**
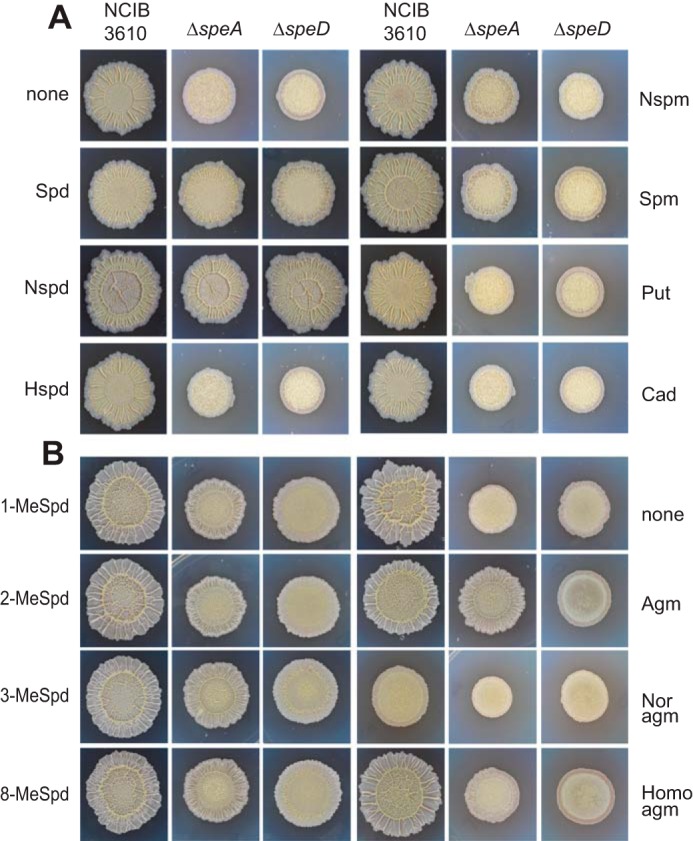
**The effect of exogenous polyamines on biofilm formation of polyamine auxotrophic strains derived from *B. subtilis* NCIB3610.** Wild-type undomesticated strain NCIB3610, gene deletion of arginine decarboxylase (strain Δ*speA*), and gene deletion of *S*-adenosylmethionine decarboxylase (strain Δ*speD*). *A*, effect of exogenous natural polyamines (100 μm) on biofilm formation. *Spd*, spermidine; *Nspd*, norspermidine; *Hspd*, homospermidine; *Nspm*, norspermine; *Spm*, spermine; *Put*, putrescine; *Cad*, cadaverine. *B*, effect of exogenous synthetic methylated spermidine analogues and agmatine structural analogues (100 μm) on biofilm formation. *1-MeSpd*, 1-methylspermidine; *2-MeSpd*, 2-methylspermidine; *3-MeSpd*, 3-methylspermidine; *8-MeSpd*, 8-methylspermidine; *Agm*, agmatine, *Noragm*, noragmatine; *Homoagm*, homoagmatine. Colony biofilms were developed on solid MSgg agar containing 100 μm natural or synthetic polyamines. Wild-type NCIB3610 colonies are ∼1 cm in diameter and the different sizes of other colonies are proportional to the wild-type.

The lack of effect of polyamine depletion on planktonic growth in *B. subtilis* is an advantage for analyzing the role of spermidine in biofilm formation, due to the absence of confounding growth-related pleiotropic responses. We previously noted that norspermidine but not homospermidine can replace spermidine in biofilm formation of the undomesticated *B. subtilis* strain NCIB3610 (18). Diverse diamines, triamines, and tetraamines (Fig. 1*A*) at a concentration of 100 μm were assessed for their ability to replace spermidine in biofilm formation of the NCIB3610 parental strain (Table 1) and arginine decarboxylase (Δ*speA*) and *S*-adenosylmethionine decarboxylase (Δ*speD*) gene deletion strains (Figs. 1*B* and 2*A*). Only norspermidine was able to replace spermidine, whereas diamines putrescine and cadaverine, and triamine homospermidine had no discernible effect on biofilm formation, confirming previous findings (17, 18). Not shown is the effect of 1,3-diaminopropane, which like putrescine and cadaverine was unable to restore biofilm formation.

**Table 1:** **Bacterial strains used in this study**

Strain	Relevant genotype/description	Source/construction*^a,b^*
NCIB3610	Prototroph	BGSC
168	*trpC2*	BGSC
JH642	*trpC2 pheA1*	67
NRS1858	JH642 *sinR*::*kan*	63
NRS1964	JH642 *amyE*::*P_hyspank_-slrR-lacI* (*spc*)	65
NRS2097	3610 *bslA*::*cat*	63
NRS2241	168 *sacA*::*P_epsA_-gfpmut2* (*kan*)	65
NRS2242	3610 *sacA*::*P_epsA_-gfpmut2* (*kan*)	65
NRS2388	168 *sacA*::*P_tapA_-gfpmut2* (*kan*)	65
NRS2394	3610 *sacA*::*P_tapA_-gfpmut2* (*kan*)	65
NRS2415	3610 *tasA*::*spc*	32
NRS3089	3610 *speA*::*spc*	17
NRS3972	3610 *speA*::*spc sacA*::*P_tapA_-gfpmut2* (*kan*)	SPP1 NRS2241 → NRS3089
NRS3974	3610 *speA*::*spc sacA*::*P_tapA_-gfpmut2* (*kan*)	SPP1 NRS2388 → NRS3089
NRS4005	3610 Δ*speD*	18
NRS4015	3610 Δ*speD sacA*::*P_epsA_-gfpmut2* (*kan*)	SPP1 NRS2241 → NRS4005
NRS4100	3610 Δ*speD sacA*::*P_tapA_-gfpmut2* (*kan*)	SPP1 NRS2388 → NRS4005
NRS5053	3610 Δ*speD sacA*::*P_tapA_-gfpmut2* (*kan*) *amyE*::*P_hyspank_-slrR-lacI* (*spc*)	SPP1 NRS1964 → NRS4100
NRS5330	3610 Δ*speD sinR*::*kan*	SPP1 NRS1858 → NRS4005
NRS5331	3610 Δ*speD amyE*::*P_hyspank_-slrR-lacI* (*spc*)	SPP1 NRS1964 → NRS4005
NRS5332	3610 *sinR*::*kan*	SPP1 NRS1858 → NCIB3610
NRS5334	3610 Δ*speD sacA*::*P_epsA_-gfpmut2* (*kan*) *amyE*::*P_hyspank_-slrR-lacI* (*spc*)	SPP1 NRS1964 → NRS4015
NRS5337	3610 *sacA*::*P_epsA_-gfpmut2* (*kan*) *amyE*::*P_hyspank_-slrR-lacI* (*spc*)	SPP1 NRS1964 → NRS2242
NRS5338	3610 *sacA*::*P_tapA_-gfpmut2* (*kan*) *amyE*::*P_hyspank_-slrR-lacI* (*spc*)	SPP1 NRS1964 → NRS2394

*^a^* BGSC represents the *Bacillus* Genetic Stock Centre.

*^b^* Direction of strain construction is indicated with phage (SPP1) → recipient strain.

A very small effect was detectable with norspermine and spermine but only in the Δ*speA* and not the Δ*speD* strain. These results confirm that only norspermidine can replace the role of spermidine in robust biofilm formation (17, 18). Norspermidine contains only aminopropyl moieties, and homospermidine only aminobutyl moieties, suggesting that the aminopropyl side of spermidine may be the active part of the structure for biofilm formation. To test this hypothesis, synthetic *C*-methylated analogues (Fig. 1*C*) of spermidine were tested (at 100 μm) to determine whether they were able to replace spermidine for biofilm formation (Fig. 2*B*). All *C*-methylated spermidine analogues were able to generate complexity (biofilm wrinkliness and three dimensionality) in biofilm formation in the Δ*speA* strain but much less so in the Δ*speD* strain. Furthermore, the 1- and 2-methylspermidine analogues were less effective than the 3- and 8-methylspermidine analogues, indicating that *C*-methylation on the aminopropyl side is more deleterious than on the aminobutyl side.

**Figure 3.**
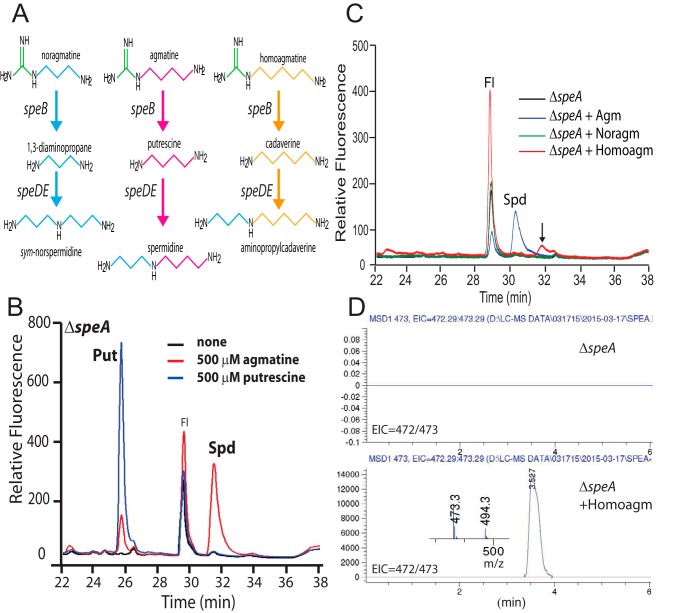
**Proposed biosynthesis of agmatine analogue-derived polyamines in *B. subtilis* and results of precursor feeding in the Δ*speA* strain.** *A*, proposed pathway for conversion of agmatine structural analogues to diamines and triamines. *B*, exogenous (500 μm) agmatine but not putrescine is converted to spermidine in Δ*speA* as detected by HPLC. *Fl*, fluorescent label; *Put*, putrescine; *Spd*, spermidine. *C*, growth of Δ*speA* with exogenous (100 μm) homoagmatine (*Homoagm*) but not noragmatine (*Noragm*) results in the production of a new peak (shown by *vertical*) *arrow* in the HPLC detection of polyamines. *Fl*, fluorescent label; *Spd*, spermidine. *D*, LC-MS analysis of Δ*speA* with or without growth in 100 μm homoagmatine (*Homoagm*). Extracted ion chromatograms (472/473) for the detection of protonated tribenzoylated aminopropylcadaverine (*m*/*z* 473.3). The mass spectrum (*inset in lower panel*) of the peak at 3.527 min, found only after growth with homoagmatine, reveals masses for protonated tribenzoylated aminopropylcadaverine (*m*/*z* 473.3) and its sodium adducted form (*m*/*z* 494.3).

Exogenous agmatine restored biofilm formation to the Δ*speA* strain, whereas putrescine did not (Fig. 2, *A* and *B*). Agmatine is the product of SpeA activity and precursor to putrescine biosynthesis in *B. subtilis* (Fig. 1*B*). The SpeB, SpeD, and SpeE enzymes of *B. subtilis* convert agmatine to putrescine and then spermidine (Figs. 1*B* and 3*A*) (36, 37). It is surprising therefore that exogenous putrescine could not restore biofilm formation to the Δ*speA* strain (Fig. 2*A*). To determine whether putrescine was converted to spermidine in the Δ*speA* strain, the strain was grown in liquid MSgg containing 500 μm putrescine, agmatine, or neither (Fig. 3*B*). Analysis of polyamine content of the cells by HPLC revealed that no spermidine is produced from exogenous putrescine, whereas it is produced from exogenous agmatine. Although the cells were washed four times with fresh polyamine-free MSgg growth medium before extraction of intracellular polyamines with trichloroacetic acid, a large peak of putrescine was present when Δ*speA* cells had been grown in exogenous putrescine. With Gram-negative bacteria, such washing is sufficient to remove externally bound polyamine, but it is feasible that the thicker envelope of Gram-positive bacteria may bind polyamines more avidly. Alternatively, putrescine may be taken up but for whatever reason not converted to spermidine. It is significant that *B. subtilis* does not encode homologues of either the *E. coli potABCD* spermidine-preferential uptake transporter or the *potFGHI* putrescine-specific uptake transporter.

There has been some confusion recently about which polyamines are synthesized by *B. subtilis* (38), in particular norspermidine. It is clear that *B. subtilis* does not encode the genes for 1,3-diaminopropane biosynthesis (18) but it is formally possible that the SpeD/SpeE route (Fig. 1*B*) for spermidine biosynthesis could aminopropylate nonnative diamines to form alternative triamines. Although *B. subtilis* does not appear to take up putrescine or alternatively, does not convert it to spermidine, the obvious uptake of agmatine and its subsequent conversion to spermidine offered an opportunity to determine whether agmatine analogues could be used to overcome the lack of a diamine uptake transporter. We hypothesized that if agmatine could be taken up and converted to putrescine by SpeB (agmatine ureohydrolase) and subsequently to spermidine, then noragmatine or homoagmatine (Fig. 3*A*) might be converted by SpeB to 1,3-diaminopropane or cadaverine, respectively; and then to norspermidine or aminopropylcadaverine, respectively, by SpeD and SpeE (Fig. 3*A*). To explore this hypothesis we synthesized 3-aminopropylguanidine (noragmatine) and 5-aminopentylguanidine (homoagmatine) (Fig. 1) and grew NCIB3610, Δ*speA* and Δ*speD* in MSgg medium supplemented with 100 μm of the agmatine analogues. Agmatine fully restored biofilm development to the Δ*speA* strain (Fig. 2*B*), and homoagmatine partially restored it. However, the biofilm defect of the Δ*speA* strain was severely exacerbated with noragmatine and even wild-type (NCIB3610) biofilm development was markedly inhibited by noragmatine. This is likely due to an observed inhibition of planktonic cell growth by exogenous noragmatine (results not shown).

Polyamine content analysis of planktonically-grown Δ*speA* cells incubated with 100 μm agmatine, noragmatine, or homoagmatine showed that agmatine is converted to spermidine and that a new peak accumulates after growth with homoagmatine, but no new peak was seen with cells incubated with noragmatine (Fig. 3*C*). Cell extracts of the Δ*speA* strain grown with or without 100 μm homoagmatine were benzoylated and analyzed by LC-MS. A peak for tribenzoylated aminopropylcadaverine (Extracted Ion Chromatogram 472/473) was detected only in cells grown with homoagmatine (Fig. 3*D*), and this peak contained masses corresponding to tribenzoylated aminopropylcadaverine (*m*/*z* 473.3) and its sodium-adducted form (*m*/*z* 494.3). These data show that homoagmatine is taken up by *B. subtilis* and converted to the triamine aminopropylcadaverine. The less effective restoration of the Δ*speA* biofilm defect by homoagmatine compared with agmatine may be due to the smaller amount of aminopropylcadaverine produced from homoagmatine compared with the amount of spermidine produced from agmatine. These data would suggest that aminopropylcadaverine can replace the function of spermidine in biofilm formation. However, noragmatine is either not taken up, or not converted to 1,3-diaminopropane, and considering that diamines are not taken up, together these data suggest that it is very unlikely that *B. subtilis* is capable of synthesizing norspermidine through the endogenous spermidine biosynthetic pathway. The inhibitory effect of noragmatine on wild-type planktonic growth and biofilm formation is not related to the production of either 1,3-diaminopropane or norspermidine because no detectable polyamines were produced from growth of Δ*speA* with noragmatine (Fig. 2*C*).

### The spermidine-dependent transcriptome

To identify cellular processes dependent on the presence of spermidine, we analyzed differences in the *B. subtilis* NCIB3610 and Δ*speD* transcriptomes at the mid-exponential phase of planktonic growth using whole genome Affymetrix microarrays. The experiments were performed in parallel in biological triplicate (hereafter “set 123” (supplemental Table S1)) and then the experiment was repeated again (after 1 week) with fresh cultures (hereafter “set 456” (supplemental Table S2)). Only genes that were more than 2-fold repressed in both sets (123 and 456) are shown in Table 2. A single gene was up-regulated more than 2-fold in both the 123 and 456 datasets: *yycC*, a gene of unknown function in the TnrA regulon that is known to be repressed by TnrA (39).

**Table 2:** **Genes with repressed steady-state levels of RNA transcripts in the *B. subtilis* Δ*speD* strain from mid-log phase of growth in polyamine-free liquid MSgg medium** The fold-repression represents the average of two independent experiments (sets 1, 2, 3 and 4, 5, 6). Each experiment analyzed three independent parallel samples (NCIB3610 compared with Δ*speD*) for a total, across the two experiments, of six microarrays each for the wild-type (parental) NCIB3610 and Δ*speD* strains.

Gene	-ve Fold-change	Functions	Regulon
*yvqI* (*liaI*)	6.84	Membrane anchor for LiaH	LiaR
*yvqH* (*liaH*)	6.82	Phage shock protein A paralogue	LiaR
*ykoL*	5.52	Unknown function, in operon with *ykzB*, induced by absence of good nitrogen sources	TnrA
*yurC* (*pucD*)	3.59	Xanthine dehydrogenase, purine catabolism	PucR
*yurD* (*pucC*)	3.59	Xanthine dehydrogenase, purine catabolism	PucR
*nasA*	3.54	Nitrate uptake transporter	TnrA
*yurF* (*pucA*)	3.48	Xanthine dehydrogenase, purine catabolism	PucR
*ywrD*	3.41	Unknown, expressed in absence of good nitrogen source	TnrA
*yurB* (*pucE*)	3.41	Xanthine dehydrogenase, purine catabolism	PucR
*cotN* (*tasA*)	3.33	Protein fiber-forming component of biofilm matrix	SinR, AbrB, RemA
*nasC*	3.33	Nitrate reductase catalytic subunit, induced under nitrogen limiting aerobic conditions	TnrA
*nasB*	3.27	Nitrate reductase catalytic subunit, induced under nitrogen limiting aerobic conditions	TnrA
*sipW*	3.19	Bifunctional signal peptidase, processes TapA and TasA components of biofilm	SinR, AbrB, RemA
*yqxM* (*tapA*)	2.88	Anchor/assembly protein for TasA fibers biofilm component	SinR, AbrB, RemA, LutR
*yccC* (*ansZ*)	2.88	l-Asparaginase	TnrA, CodY
*yveP* (*epsF*)	2.83	Glucosyltransferase family 1, biofilm exopolysaccharide synthesis	SinR, AbrB, RemA
*yveR* (*epsH*)	2.79	Synthesis poly-*N*-acetylglucosamine, UDP-*N*-acetylglucosamine transferase	SinR, AbrB, RemA, EAR
*yveQ* (*epsG*)	2.69	Unknown function, biofilm formation	SinR, AbrB, RemA
*yrbD*	2.66	Sodium/proton dependent alanine uptake transporter	TnrA
*yveS* (*epsI*)	2.64	Synthesis of poly-*N*-acetylglucosamine, polysaccharide pyruvyl transferase	SinR, AbrB, RemA
*yoaB*	2.63	2-oxoglutarate-proton symporter, induced by methionine starvation	S-box
*yveT* (*epsJ*)	2.62	Glucosyltransferase family 1, biofilm exopolysaccharide synthesis	SinR, AbrB, RemA, EAR
*yocE* (*des*)	2.60	Phospholipid desaturase, adaptation of membrane fluidity at low temperatures	DesR
*yunJ* (*pucJ*)	2.58	Uric acid permease, purine utilization	PucR, TnrA
*ykrT* (*mtnK*)	2.55	Methylthioribose kinase, methionine salvage	S-box
*yveN* (*epsD*)	2.47	Unknown function, glycosyltransferase 1 family	SinR, AbrB, RemA, EAR
*yvfD* (*epsM*)	2.44	Unknown function, transferase hexapeptide repeat family	SinR, AbrB, RemA
*yitJ*	2.44	Methionine synthase	S-box
*yveK* (*epsA*)	2.43	Unknown, putative modulator of EpsB activity	SinR, AbrB, RemA
*yunK* (*pucK*)	2.43	Uric acid permease, purine utilization	PucR, TnrA
*yvfC* (*epsL*)	2.42	Similar to UDP-galactose phosphate transferase	SinR, AbrB, RemA
*yvfA* (*epsK*)	2.39	Poly-*N*-acetylglucosamine exporter	SinR, AbrB, RemA, EAR
*yveO* (*epsE*)	2.34	Glycosyltransferase and inhibitor of motility, clutch protein	SinR, AbrB, RemA, EAR
*yvfE* (*epsN*)	2.28	Unknown	SinR, AbrB, RemA, EAR
*yunL* (*pucL*)	2.14	Uricase, expressed in absence of good nitrogen source or presence of purines	PucR, TnrA
*yveL* (*epsB*)	2.13	Protein tyrosine kinase	SinR, AbrB, RemA
*metC* (*metE*)	2.08	Cystathionine β-lyase, methionine biosynthesis	S-box
*yvfF* (*epsO*)	2.00	Synthesis of poly-*N*-acetylglucosamine, polysaccharide pyruvyl transferase	SinR, AbrB, RemA
*yveJ* (*slrR*)	1.98	Antagonist of SlrA and SinR, transcriptional activator of sporulation, competence	Abh, AbrB, SinR

Genes that were more than 2-fold repressed in both the 123 and 456 datasets indicated that the block in flux of methionine and *S*-adenosylmethionine into spermidine in the Δ*speD* strain was sensed by the cell. There was a marked repression of genes in the PucR, TnrA, and S-box regulons, suggesting that nitrogen sources and *S*-adenosylmethionine were present in excess in the Δ*speD* strain. Genes involved in purine catabolism (40), including xanthine dehydrogenase (*pucACDE*), uric acid permease (*pucJK*), and uricase (*pucLM*) in the PucR regulon, which is itself in the TnrA regulon were repressed, again indicating nitrogen excess. The regulator PucR was shown previously to induce expression of the *pucJKLM* operon and to repress expression of the *pucABCDE* operon (41), however, in the Δ*speD* strain both operons were repressed. Other TnrA-regulated genes that were repressed in the Δ*speD* strain were the divergently transcribed nitrate uptake transporter *nasA* and the nitrate reductase catalytic subunits *nasB* and *nasC*, further suggesting nitrogen excess. The TnrA-regulated genes encoding a sodium/proton-dependent alanine uptake transporter (*yrbD*), l-asparaginase (*ansZ*), and proteins of unknown function *ywrD* and *ykoL*, which are normally induced in the absence of a good nitrogen source, were all repressed in the Δ*speD* strain.

Genes in the S-box regulon responsible for methionine biosynthesis were repressed: cystathionine β-lyase (*metC*) and methionine synthase (*yitJ*), as well as genes usually induced by methionine starvation, 2-oxoglutarate-proton symporter (*yoaB*) and a xylulose kinase-like protein (*yoaC*). In addition, S-box-regulated genes involved in methionine salvage (42) from the decarboxylated *S*-adenosylmethionine-derived methylthioadenosine coproduct of spermidine synthase activity were repressed: 5-methylthioribose-1-phosphate isomerase (*mtnA*) and methylthioribose kinase (*mtnK*). The genes that were most repressed were the *liaIH* genes encoding a phage shock protein homologue (*liaH*) and its membrane anchor (*liaI*), which are thought to be induced by cell envelope stress and are suggested to protect membrane integrity (43). It may be relevant to the repression of *liaHI* expression that another repressed gene in the Δ*speD* strain encodes a phospholipid desaturase (*des*) that is repressed by increased membrane fluidity (44).

In addition to transcriptome changes in the Δ*speD* strain that reflect nitrogen and methionine excess, and potentially membrane fluidity changes, the other significant change to the transcriptome of the Δ*speD* strain is the repression of multiple members of the *epsA-O* and *tapA-sipW-tasA* operons that are required for production of the exopolysaccharide and the fibrous TasA protein that form essential components of the biofilm matrix. It is particularly notable that transcription of the SinR antagonist *slrR* is also repressed (although only 1.9-fold in the 123 data set and 2.0-fold in the 456 data set, so it did not quite make the 2.0-fold repressed in both data sets criterion).

### Spermidine depletion reduces the number of cells activating matrix gene expression

**Figure 4.**
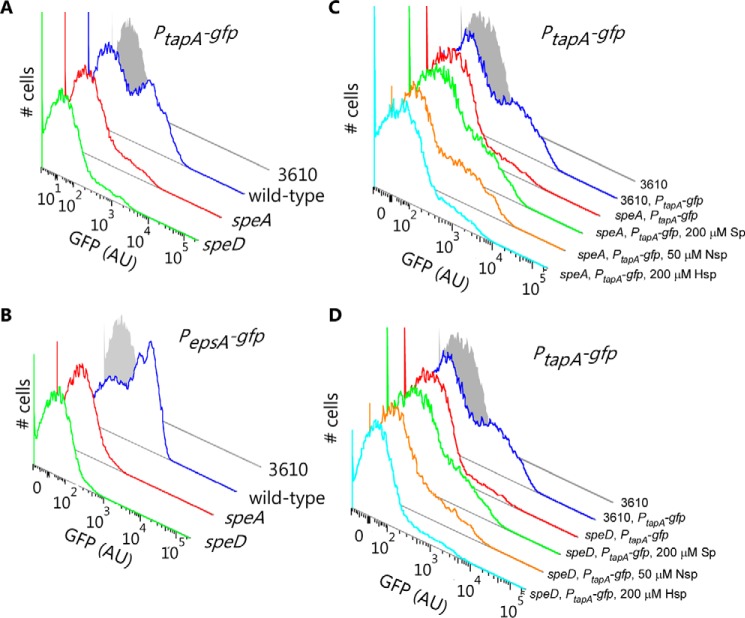
**Expression of both the *tapA-sipW-tasA* and *eps(A-O*) operons is reduced in spermidine auxotrophic colony biofilms.** Flow cytometry analysis of single cells harvested from complex colonies grown at either 37 °C for 17 h (*A* and *B*) or 30 °C for 40 h (*C* and *D*). *A*, *C*, and *D*, expression from the *tapA* promoter; *B*, expression from the *epsA* promoter. Strains analyzed were: NCIB3610, NRS2242 (3610, *sacA*::*P_epsA_-gfp*), NRS3972 (*speA*::*spc*, *sacA*::*P_epsA_-gfp*), NRS2394 (3610, *sacA*::*P_tapA_-gfp*], NRS4100 (Δ*speD*, *sacA*::*P_tapA_-gfp*] and NRS3974 (*speA*::*spc*, *sacA*::*P_tapA_-gfp*). *C* and *D*, the exogenous addition of spermidine (*Sp*), norspermidine (*Nsp*) or homospermidine (*Hsp*) to the growth medium is indicated. The NCIB3610 non-fluorescent control sample shown in *C* and *D* is the same, as these experiments were run simultaneously.

Transcriptome analysis of the Δ*speD* strain indicated that the *epsA-O* and *tapA-sipW-tasA* operons were repressed in the absence of spermidine. These operons exhibit a bimodal transcription profile that is a consequence of a dynamic and complex regulatory network (45, 46). To verify the data generated by the transcriptome analysis and to determine whether: 1) transcript levels per cell, or 2) the number of cells activating transcription of the operons, were reduced in the absence of spermidine, we used flow cytometry (FACS)-based single cell transcriptional analysis. The promoter regions of P*_eps_* and P*_tapA_* were fused to the open reading frame encoding green fluorescent protein (*gfp*) and integrated into an ectopic chromosomal location (*sacA*) to generate transcriptional reporter fusion strains (Table 1). Subsequent flow cytometry analysis of cells grown under biofilm formation conditions revealed that transcription from both the P*_eps_* and P*_tapA_* promoters was decreased in the spermidine auxotrophs due to a reduction in the number of cells activating matrix gene expression (Fig. 4).

The ability of each triamine to recover transcription of the *tapA-sipW-tasA* matrix operon in the Δ*speA* and Δ*speD* strains was examined. Exogenous spermidine or norspermidine but not homospermidine maintained transcription from the P*_tapA_* promoters (Fig. 4). Consistent with the greater effectiveness of norspermidine *versus* spermidine in restoring biofilm formation to the Δ*speA* and Δ*speD* strains (18), lower concentrations of norspermidine compared with spermidine were required to maintain transcription from the biofilm matrix operons (Fig. 4). These data indicate that in the wild-type strain endogenously-synthesized spermidine is required for expression of the biofilm matrix operons.

### Modifying slrR or sinR levels controls matrix gene expression and restores biofilm formation to spermidine auxotrophs

**Figure 5.**
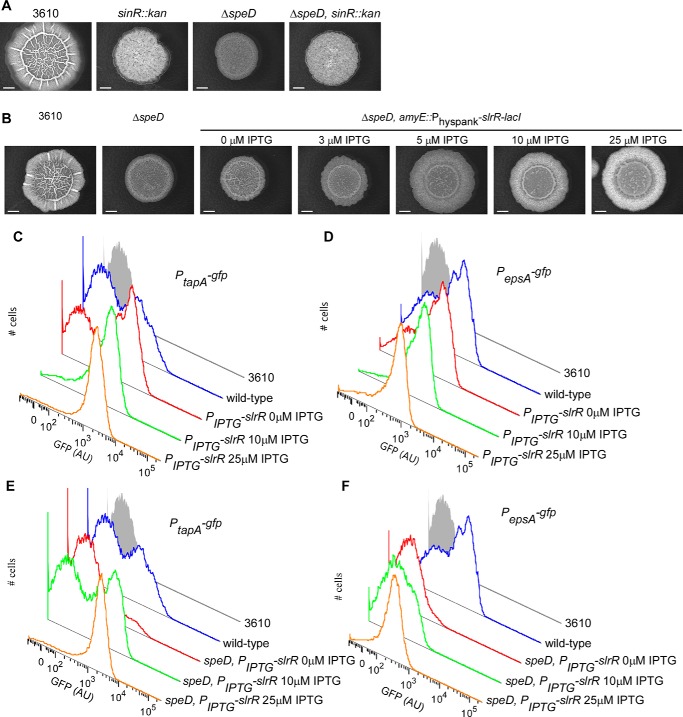
**Restoration of *slrR* expression in the *speD* mutant results in restoration of colony biofilm complexity and expression of both the *eps(A-O*) and *tapA-sipW-tasA* operons.** *A*, disruption of *sinR* results in a hyper-complex complex colony in both an otherwise wild-type strain and in the Δ*speD* mutant. Biofilms were grown at 30 °C for 48 h prior to imaging. Strains included: NCIB3610, NRS5332 (*sinR*::*kan*), NRS4005 (Δ*speD*), and NRS5330 (Δ*speD, sinR*::*kan*). *B*, restoration of *slrR* expression in Δ*speD* mutant restores colony biofilm complexity. Expression of *slrR* was induced by the addition of IPTG to the MSgg agar, and complex colonies were grown at 30 °C for 48 h prior to imaging. Strains included: NCIB3610, NRS4005 (Δ*speD*) and NRS5331 (Δ*speD, amyE*::*P_hyspank_-slrR-lacI*). *C-F,* heterologous expression of *slrR* in the wild-type (*C*, *D*) or *speD* mutant (*E*, *F*) results in unimodal expression of both the *tapA-sipW-tasA* (*C*, *E*) and *eps(A-O*) (*D*, *F*) operons. Flow cytometry analysis of single cells harvested from complex colonies grown at 37 °C for 17 h, either in the absence of IPTG induction or with induction of *slrR* expression with the addition of 10 or 25 μm IPTG. Strains analyzed were: NCIB3610, NRS2394 (3610, *sacA*::*P_tapA_-gfp*), NRS5338 (3610, *sacA*::*P_tapA_-gfp amyE*::*P_hyspank_-slrR-lacI*), NRS5337 (3610, *sacA*::*P_epsA_-gfp amyE*::*P_hyspank_-slrR-lacI*), NRS5053 (Δ*speD*, *amyE*::*P_hyspank_-slrR-lacI*, *sacA::P_tapA_-gfp*), NRS2242 (3610, *sacA::P_epsA_-gfp*), and NRS5334 (Δ*speD*, *amyE*::*P_hyspank_-slrR-lacI*, *sacA*::*P_epsA_-gfp*). In *C–F* the NCIB3610 sample is the same in all four graphs as experiments were run simultaneously. In *C* and *E* the same wild-type sample is shown in both for ease of comparison with other samples. In *D* and *F* the same wild-type sample is shown in both to facilitate comparison.

The transcriptome analysis provided a clue why expression of the *epsA-O* and *tapA-sipW-tasA* was reduced in the Δ*speD* strain: the level of the *slrR* transcript was reduced 2-fold. SlrR functions as an activator of *epsA-O* and *tapA-sipW-tasA* expression by binding to the transcriptional repressor SinR (47), preventing SinR from binding to the promoters of the *epsA-O* and *tapA-sipW-tasA* operons. We predicted that the requirement for spermidine in biofilm formation could be bypassed by: 1) deleting the repressor *sinR*; or 2) ectopically expressing the SinR antagonist SlrR. To test these predictions we deleted the *sinR* coding region in the wild-type parental NCIB3610 strain and in the Δ*speD* strain, and then assessed biofilm formation. The *sinR* deletion (Δ*sinR*) strain forms tight, highly wrinkled macrocolonies that are a consequence of deregulated expression of the biofilm matrix operons and lack of motility (45) (Fig. 5*A*). Deletion of *sinR* in the Δ*speD* background (Δ*sinR* Δ*speD*) produced the same highly wrinkled morphology as Δ*sinR*, indicating that deletion of *sinR* circumvented the spermidine requirement for biofilm formation. Furthermore, we introduced the *slrR* open reading frame into the Δ*speD* strain at the *amyE* chromosomal locus under control of an IPTG-inducible promoter. Increasing expression of *slrR* in the Δ*speD* background by incubation of the strain with increasing concentrations of IPTG^5^ resulted in a correspondingly more wrinkled, complex biofilm (Fig. 5*B*). Additionally, we introduced the same *slrR* construct into strains carrying the P*_eps_* and P*_tapA_* promoter-*gfp* reporter fusions to allow assessment of the impact of ecoptic expression of *slrR* on transcription of the matrix operons. Ectopic expression of *slrR* in the Δ*speD* strain resulted in an increase in P*_eps_* and P*_tapA_* expression due to more cells expressing these promoters (Fig. 5, *C–F*). This indicates that the spermidine-dependent maintenance of P*_eps_* and P*_tapA_* expression can be bypassed by ectopic expression of *slrR* in spermidine auxotrophic strains, and that ectopic expression of *slrR* results in all cells in the biofilm population expressing the matrix operons. This was observed for both wild-type and spermidine auxotrophic strains confirming that increasing the level of SlrR can bypass the requirement for spermidine in biofilm formation.

## Discussion

The physiological roles of polyamines in bacteria are poorly understood. Furthermore, it is also important to distinguish between the role of free polyamines in cell physiology and polyamines that are covalently incorporated into more complex molecules such as siderophores and other natural products. A close relative of *B. subtilis*, the anthrax agent *Bacillus anthracis*, incorporates spermidine into the stealth siderophore petrobactin (48), which is a pathogenicity determinant of the anthrax pathogen (49). Although *B. subtilis* is not known to produce spermidine-based natural products, it does have the ability to *N*-acetylate spermidine (50), a modification that would abrogate its polyamine functionality. It is therefore reasonable to assume that the role of spermidine in biofilm formation is likely to be manifested as the free triamine, interacting with unknown substrates or receptors. In eukaryotes it is well known that the essential role of spermidine in growth is dependent on transfer of the aminobutyl group of spermidine to the translation elongation factor eIF5A to form the deoxyhypusine modification. This role of spermidine cannot be replaced by the aminobutyl group of diamine putrescine (51), however, the aminobutyl group of homospermidine can be used for deoxyhypusine formation by deoxyhypusine synthase (52). In contrast, the essential role of spermidine in growth of α-proteobacterium *A. tumefaciens* C58 is determined by its 1,3-diaminopropane moiety, containing the aminopropyl group (13). The function of spermidine in growth of *A. tumefaciens* can be replaced by the simple diamine 1,3-diaminopropane, although its function in growth is unknown (13). With *B. subtilis*, we found that only triamines containing an aminopropyl moiety enable biofilm development, suggesting a specific interaction of the aminopropyl moiety with an unknown target. We noticed that exogenous putrescine was not converted to spermidine in the Δ*speA* strain, whereas exogenous agmatine was readily converted to spermidine in the same strain. Inspection of the *B. subtilis* genome revealed that there are no homologues of either the putrescine-specific (*potFGHI*) or spermidine-preferential (*potABCD*) ABC-type transporters that are present in closely related species such as *B. anthracis* and *Bacillus cereus*. The lack of conversion of exogenous putrescine to spermidine in the Δ*speA* strain is consistent with the lack of a diamine uptake transporter in *B. subtilis*. Because agmatine is taken up by *B. subtilis*, we decided to use the agmatine transporter as an alternative approach to assess whether or not 1,3-diaminopropane or cadaverine could be produced intracellularly and whether they could be aminopropylated to form norspermidine or aminopropylcadaverine, respectively. We synthesized the agmatine structural analogues noragmatine and homoagmatine and assessed their ability to restore biofilm formation to the Δ*speA* and Δ*speD* strains. Noragmatine inhibited wild-type biofilm formation and also inhibited planktonic growth; no 1,3-diaminopropane or norspermidine was detected in cells. Homoagmatine was able to partially restore biofilm complexity to the Δ*speA* but not Δ*speD* strains, and this correlated with the accumulation of aminopropylcadaverine in Δ*speA* cells.

The inability of homoagmatine to restore biofilm formation to the Δ*speD* strain is consistent with the fact that cadaverine produced from homoagmatine could not be aminopropylated in a strain lacking *speD*, suggesting that in the Δ*speA* strain aminopropylcadaverine synthesis is responsible for restoration of biofilm formation. It is notable that aminopropylcadaverine and homospermidine both possess eight methylene carbons but only the analogue containing an aminopropyl group, *i.e.* aminopropylcadaverine, was able to function in biofilm formation. The fact that spermidine, norspermidine, and aminopropylcadaverine can restore biofilm formation to the Δ*speA* strain indicates that the length and symmetrical status of the triamine are not important for its function in biofilm formation. However, the presence of an aminopropyl or 1,3-diaminopropane moiety is critical. Together, these observations suggest that in the wild-type strain, a rather specific interaction must occur between the aminopropyl side of spermidine and one or more select targets that may be sensors, receptors, or specific proteins that require spermidine to function. It should be noted that we do not know whether the site of action of endogenously-produced spermidine in biofilm development is intra- or extracellular.

### Transcriptome response to spermidine auxotrophy

The two main transcriptome responses in the Δ*speD* strain concern metabolism and production of the biofilm matrix. Relative to the wild-type strain, the Δ*speD* strain has down-regulated pathways involved in purine catabolism, methionine salvage, and methionine and nitrogen starvation responses. These responses are part of the PucR, TnrA, and S-box regulons. Elimination of *S*-adenosylmethionine decarboxylase (SpeD) activity blocks flux of methionine and nitrogen into spermidine via *S*-adenosylmethionine. The transcriptome responses suggest that the cell senses excess *S*-adenosylmethionine, methionine, and nitrogen generally. Furthermore, the methionine salvage pathway that rescues methionine and the purine component from methylthioadenosine generated by spermidine biosynthesis is down-regulated. This may contribute also to the purine catabolism/uptake response, which down-regulates enzymes and transporters involved in salvaging purines and nitrogen. Although this metabolic response to a presumed methionine/*S*-adenosylmethionine excess is unsurprising, it is an interesting insight into the extent of nitrogen flux through these metabolites into spermidine. Two unrelated genes affecting membrane fluidity were repressed in Δ*speD*, the *liaIH* operon, encoding membrane anchor and phage shock protein A paralogue, and the *des* gene encoding a phospholipid desaturase whose expression increases membrane fluidity (53) and is induced at low temperatures. The phage shock protein A paralogue LiaH is produced in response to cell envelope perturbations and is located in the membrane (43). Repression of *liaHI* and *des* expression suggests that the membrane of the Δ*speD* strain may be sensed by the cell as being too fluid.

Microarray detection of repression of the *epsA-O* operon encoding the proteins required to produce and secrete the biofilm matrix exopolysaccharide, and the *tapA-sipW-tasA* operon required to produce and anchor the fibrous matrix protein TasA, in the Δ*speD* strain explains at one level why spermidine depletion prevents robust biofilm formation. Repression of the *slrR*-encoded biofilm regulatory protein means that the biofilm repressor SinR would not be inhibited by its normal binding to SlrR, and therefore unbound SinR would be more able to repress biofilm formation. Using single cell gene expression analysis, we demonstrated that spermidine and norspermidine but not homospermidine restored biofilm formation and expression of the *epsA-O* and *tapA-sipW-tasA* operons. The decrease in expression of the matrix operons detected in the microarray analysis was due to a decrease in the number of cells expressing the matrix operons rather than a decrease in the rate of transcription per cell. Spermidine depletion therefore decreases the number of cells expressing the biofilm matrix operons. Ectopic expression of *slrR* restored biofilm formation to the Δ*speD* strain, and increased the number of cells expressing the matrix operons.

Expression of *slrR* is known to be positively regulated by the DNA-binding protein RemA, and the sites bound by RemA that are necessary for activation of *slrR* expression can also be partially bound by SinR, thereby occluding the RemA-binding sites (54). Deletion of *sinR* resulted in a highly wrinkled dense biofilm phenotype in the wild-type 3610 strain and a similar morphology resulted from its deletion in the Δ*speD* strain. Ectopic expression of *slrR* or deletion of *sinR* in the Δ*speD* strain restores biofilm formation. This effect of spermidine depletion on *slrR* expression, and the fact that deletion of *sinR* or ectopic expression of *slrR* in the spermidine-depleted mutant strain restores biofilm formation is reminiscent of the role of the phosphodiesterase YmdB in biofilm formation (55, 56). Expression of *slrR* is dependent on the activity of YmdB but unlike the effect of spermidine-depletion, deletion of *ymdB* activates motility genes, especially *hag*. Activation of biofilm matrix operon expression in the Δ*speD* strain when *sinR* is deleted suggests that spermidine depletion affects the activity of SinR rather than RemA, because deletion of *sinR* allows activation of matrix operon expression, indicating that RemA is likely to be active. However, we did not detect up-regulation of motility-related genes such as the flagellin *hag* gene, which might be expected if SinR was active, in the Δ*speD* strain. Levels of *hag* transcript detected by the microarray analysis were changed by less than 10% in the Δ*speD* strain (in fact a decrease). In the domesticated *B. subtilis* 168 strain, proteomic analysis of a *speD* deletion derivative identified 10 proteins exhibiting altered abundance, of which two were more abundant: the Hag protein and MetK (methionine synthase) (37), whereas we did not detect up-regulation of *hag* and we detected repression of the *yitJ* methionine synthase. The only agreement between these two analyses was that TasA protein and *tasA* transcript were repressed in a Δ*speD* strain of *B. subtilis* 168 and NICB3610, respectively.

The master regulator of SinR activity is the phosphorylated form of the Spo0A transcription factor, which is regulated by a complex of kinases and phosphorelay proteins that forms a nexus for integrating diverse environmental, nutritional, and physiological signals (21, 22). Whether spermidine is required for function of this regulatory complex is not known. However, it is known that deletion of spermidine synthase (*speE*) severely delays sporulation in *B. subtilis* 168 (57), and pharmacological inhibition of arginine decarboxylase (SpeA) also results in partial depletion of spermidine and delayed sporulation in *B. subtilis* 168M (58). Furthermore, in *B. subtilis* the spermidine *N*-acetyltransferase PaiA, the activity of which reduces spermidine levels, is implicated in the negative regulation of sporulation (59). Master regulator of sporulation Spo0A (60, 61) is also required for biofilm formation (35), although different kinases phosphorylate Spo0A to different levels to instigate biofilm (low threshold levels of Spo0A-P) or sporulation developmental programs (high threshold values) (21, 22). Within a biofilm, sporulating cells arise from matrix-producing cells, suggesting a sequence of low to high levels of phosphorylated Spo0A (62). The requirement for spermidine in sporulation and biofilm formation indicates that spermidine acts upstream of Spo0A phosphorylation to enable normal initiation of developmental programs. The effects of spermidine deficiency could be construed as a dampening of the stochastic/noisy processes that influence the amount of Spo0A-P produced. However, it cannot be ruled out that spermidine affects sporulation and biofilm formation downstream of Spo0A in different, independent manners.

## Experimental procedures

### Bacterial strains and growth conditions

All *B. subtilis* strains used in this study are listed in Table 1. For routine growth *B. subtilis* was cultured in liquid LB (10 g of NaCl, 5 g of yeast extract, 10 g of tryptone per liter) or on LB plates solidified with 1.5% (w/v) select agar (Invitrogen) at 37 °C. For growth in polyamine-free medium, *B. subtilis* was grown in liquid MSgg (5 mm potassium phosphate and 100 mm MOPS at pH 7.0 supplemented with 2 mm MgCl_2_, 700 μm CaCl_2_, 50 μm MnCl_2_, 50 μm FeCl_3_, 1 μm ZnCl_2_, 2 μm thiamine, 0.5% glycerol, and 0.5% glutamate) (31), or on MSgg plates solidified with 1.5% select agar. Antibiotics were used at the following concentrations as required: 100 μg/ml of spectinomycin, 5.0 μg/ml of chloramphenicol, and 10 μg/ml of kanamycin. Standard polyamines were obtained from Sigma, and *sym*-homospermidine was a kind gift from Patrick Woster (Medical University of South Carolina).

### Strain construction

All *B. subtilis* strains created during this study are listed in Table 1. To introduce regions of interest into *B. subtilis* strain NCIB3610 and its derivatives, SPP1 phage transductions were performed as previously described (63).

### Colony biofilm formation

Strains of *B. subtilis* were grown overnight on MSgg plates at 37 °C. Three-ml aliquots of MSgg liquid broth samples were inoculated with individual colonies and grown at 37 °C with shaking for 6 h. Ten μl of the cell culture was placed onto MSgg plates and incubated at 30 °C for 48 h for morphology studies or at 37 °C for 17 h for flow cytometry studies. For growth of colony biofilms in the presence of polyamine analogues, *B. subtilis* strains were grown overnight on MSgg plates at 37 °C. Single colonies were inoculated into 3 ml of MSgg and grown with shaking at 37 °C for 6 h. To set up complex colonies, 10 μl of cells were spotted on MSgg plates supplemented with different concentrations of polyamines or analogues and incubated for 48 h at 30 °C before imaging. Images of colony biofilms were recorded using a Nikon D3200 digital camera mounted on a Kaiser RS3XA copy stand.

### RNA preparation for microarray analysis

Single colonies of *B. subtilis* NCIB3610 and the derived Δ*speD* strain grown on solid MSgg agar plates were used to inoculate 25 ml of fresh MSgg liquid medium in 250-ml flasks and grown with shaking at 37 °C overnight. The *A*_600 nm_ of the overnight cultures was determined and appropriate aliquots were used to inoculate 25 ml of fresh MSgg in 250-ml flasks such that the starting *A*_600 nm_ was 0.01. These cultures were then grown to an *A*_600 nm_ of 0.5. This subculturing process was undertaken to ensure that any contaminating spermidine would be diluted out of the Δ*speD* strain. Aliquots of 1.5 ml of the 0.5 *A*_600 nm_ cultures were then transferred to 4 microcentrifuge tubes and centrifuged to pellet the bacterial cells. Each pellet was resuspended in 175 μl of RNAwiz (from an Ambion Ribopure kit). The contents of pairs of tubes were combined into single tubes containing glass beads. These were then vortexed, centrifuged, and the lysates combined into a single tube for each sample. RNA was prepared from the lysates according to the Ambion Ribopure kit instructions and purified RNA was stored at −20 °C until DNase treatment, which was also performed according to the Ambion Ribopure kit instructions. The integrity of the RNA and effectiveness of the DNase treatment was confirmed by analyzing the RNA samples on an agarose gel. Equal quantities of RNA for each sample were then deposited with the UT Southwestern Microarray Core facility for analysis by Affymetrix *B. subtilis* whole genome microarrays. Microarray data were analyzed with Partek software and the normalization method employed was MAS5. Statistical analysis was performed using unpaired *t* test. The GEO accession number for the microarray data is GSE96942.

### Polyamine analysis by HPLC and LC-MS

Cells of *B. subtilis* NCIB3610 were grown in MSgg medium with or without exogenous amines then centrifuged in 1.5-ml microcentrifuge tubes (18,000 × *g*, 3 min) and the pellet washed several times. Bacterial pellets were resuspended in MOPS lysis buffer (20 mm MOPS, pH 8.0, 10 mm NaCl, 4 mm MgCl_2_) and the cell suspension was subjected to three cycles of freeze/thawing. Trichloroacetic acid (40%) was added to a final concentration of 10% to extract the polyamines. After centrifugation, the polyamine-containing supernatant was frozen (−80 °C) until further use. For HPLC analysis, the trichloroacetic acid extract supernatant was derivatized with a fluorescent label (AccQ-fluor, Waters), using the AccQ-Fluor reagent labeling kit, according to the manufacturer's protocol. Derivatized polyamines were resolved by HPLC using a hydrolysate amino acid analysis column as previously described (18). For analysis of polyamines by LC-MS, polyamines were benzoylated and analyzed on an Agilent Infinity LC-MS and Agilent 1100 series LC-MS with electrospray probes, using an Agilent XDB-C18 column, as previously described (64).

### Flow cytometry

The fluorescence of strains harboring *gfp* transcriptional reporter fusions was measured in single cells extracted from complex colonies grown at 37 °C for 17 h by flow cytometry and the resulting data analyzed as described previously (65). To liberate cells from the biofilm matrix, biofilm colony samples were collected and disrupted and the resulting sample subjected to gentle sonication to release cells from the matrix. During the cytometry the individual cells were detected and data from 100,000 individual cells collected. The data were combined and used to generate the histograms shown in Figs. 4 and 5. A minimum of three biological replicates were performed for each experiment.

### Synthesis of noragmatine and homoagmatine

Noragmatine dihydrobromide and homoagmatine ditosylate were synthesized from *N*-Cbz-1,3-diaminopropane hydrochloride (Aldrich) and *N*-Cbz-1,5-diaminopentane hydrochloride (Aldrich), respectively, using *N,N*′-di-Boc-*N*″-trifylguanidine (Aldrich) for guanidinylation of the amino group. Reactions were carried out in dichloromethane for 2 h at 20 °C following a general protocol of the amino group guanidinylation (66) that resulted in Cbz-di-Boc-derivatives of noragmatine and homoagmatine in almost quantitative yields. Simultaneous removal of Boc and Cbz-protecting groups with 35% HBr/AcOH afforded well crystallized dihydrobromide of noragmatine and semi-solid dihydrobromide of homoagmatine. The last was converted to well crystallized ditosylate of homoagmatine using Dowex 1-X8 resin in TosO^−^ form.

NMR spectra were registered in D_2_O on a Bruker Avance AMX III 400 (Germany) spectrometer with the working frequency of 400.1 MHz for ^1^H NMR (2,2-dimethyl-2-silapentanesulfonic acid as an internal standard) and 100.1 MHz for ^13^C (with carbon-proton decoupling, 2,2-dimethyl-2-silapentanesulfonic acid as an internal standard), chemical shifts are given in ppm (spectra for noragmatine (Gua-(CH_2_)_3_-NH_2_) and homoagmatine (Gua-(CH_2_)_5_-NH_2_) are presented in supplemental Fig. S1.

High resolution mass spectra (HRMS) were registered on a Bruker Daltonics microTOF-Q II instrument using electrospray ionization (ESI). The measurements were acquired in a negative ion mode; interface capillary voltage 3700 V; mass range from *m*/*z* 50 to 3000; external calibration (Electrospray Calibrant Solution, Fluka); nebulizer pressure 0.4 Bar; flow rate 3 μl/min; nitrogen was applied as a dry gas (4 L/min); interface temperature 190 °C. A sample of 3-aminopropylguanidine (noragmatine) dihydrobromide in 50% aqueous acetonitrile solution was injected into the mass spectrometer chamber from an Agilent 1260 HPLC system equipped with an Agilent Poroshell 120 EC-C18 (3.0 × 50 mm; 2,7 μm) column using an autosampler. Elution with a linear gradient of acetonitrile (50 → 100%) in water, flow rate of 200 μl/min, the retention time, 1.8 min. A syringe injection to mass spectrometer was used for 5-aminopentylguanidine (homoagmatine) ditosylate solution in 50% aqueous acetonitrile.

Melting points were determined in open capillary tubes on Electrothermals Mel-Temp 1202D instrument and are uncorrected. TLC was carried out on Kieselgel 60 F_254_ plates (Merck) in the system: *n*-BuOH-AcOH-Py-H_2_O, 4:2:1:2, and aminoalkylguanidines (noragmatine) and (homoagmatine) were detected on the plates by ninhydrin staining. Dihydrobromide of noragmatine: m.p. 153–154 °C (from EtOH); *R_f_* 0.52. ^1^H NMR (D_2_O) δ: 3.39 (t, 2 H, ^3^*J*_HH_ 6.9 Hz, CH_2_NH) (where the underlined H indicates the chemical shift), 3.16 (dd, 2 H, ^3^*J*_HH_ 7.8 Hz, ^3^*J*_HH_ 8.0 Hz, CH_2_NH_2_), 2.09–2.03 (m, 2 H, CH_2_CH_2_CH_2_). ^13^C NMR (D_2_O) δ: 156.93, 38.30, 36.96, 26.18. HRMS (ESI-MS): calculated for C_4_H_13_Br_2_N_4_ [M-H]^−^ 274.9481, found 276.9484. Ditosylate of homoagmatine: m.p. 192–193 °C (from EtOH); *R_f_* 0.55. ^1^H NMR (D_2_O) δ: 7.77 (d, 4 H, ^3^*J*_HH_ 8.1 Hz, Ar-H), 7.36 (d, 4 H, ^3^*J*_HH_ 8.1 Hz, Ar-H), 3.22 (t, 2 H, ^3^*J*_HH_ 7.0 Hz, CH_2_NH), 3.05 (dd, 2 H, ^3^*J*_HHa_ 7.6 Hz, ^3^*J*_HHb_ 7.7 Hz, CH_2_NH_2_), 1.70–1.62 (m, 2 H, CH_2_CH_2_NH_2_), 1.61–1.55 (m, 2 H, CH_2_CH_2_NH), 1.41–1.35 (m, 2 H, CH_2_CH_2_CH_2_CH_2_CH_2_). ^13^C NMR (D_2_O) δ: 156.75, 142.51, 139.51, 129.47, 125.40, 40.88, 39.34, 27.42, 26.36, 22.85, 20.50. HRMS (ESI-MS): calculated for C_20_H_32_N_4_O_6_S_2_ [M-H]^−^ 487.1680, found 487.1684.

## Author contributions

L. H., N. R. S.-W., and A. J. M. conceived the study and analyzed the data. L. H., B. L., J. W., A.-S. F., and S. H. K. performed experiments. J. N. performed LC-MS analysis. M. K. and A. K. synthesized polyamine and agmatine analogues. A. J. M., N. S. W., A. K., and L. H. wrote the manuscript.

## Supplementary Material

Supplemental Data

## Footnotes

5 The abbreviations used are:

IPTG: isopropyl 1-thio-β-d-galactopyranoside
HRMS: high resolution mass spectra
ESI: electrospray ionization.

## References

[1] Michael A. J. (2016) Biosynthesis of polyamines and polyamine-containing molecules. Biochem. J. 473, 2315–2329 27470594 10.1042/BCJ20160185

[2] Michael A. J. (2016) Polyamines in eukaryotes, bacteria, and archaea. J. Biol. Chem. 291, 14896–14903 27268252 10.1074/jbc.R116.734780 PMC4946907

[3] Park M. H., Cooper H. L., and Folk J. E. (1981) Identification of hypusine, an unusual amino acid, in a protein from human lymphocytes and of spermidine as its biosynthetic precursor. Proc. Natl. Acad. Sci. U.S.A. 78, 2869–2873 6789324 10.1073/pnas.78.5.2869 PMC319460

[4] Cooper H. L., Park M. H., Folk J. E., Safer B., and Braverman R. (1983) Identification of the hypusine-containing protein hy+ as translation initiation factor eIF-4D. Proc. Natl. Acad. Sci. U.S.A. 80, 1854–1857 6403941 10.1073/pnas.80.7.1854 PMC393708

[5] Schnier J., Schwelberger H. G., Smit-McBride Z., Kang H. A., and Hershey J. W. (1991) Translation initiation factor 5A and its hypusine modification are essential for cell viability in the yeast *Saccharomyces cerevisiae*. Mol. Cell. Biol. 11, 3105–3114 1903841 10.1128/mcb.11.6.3105-3114.1991 PMC360154

[6] Yanagisawa T., Sumida T., Ishii R., Takemoto C., and Yokoyama S. (2010) A paralog of lysyl-tRNA synthetase aminoacylates a conserved lysine residue in translation elongation factor P. Nat. Struct. Mol. Biol. 17, 1136–1143 20729861 10.1038/nsmb.1889

[7] Roy H., Zou S. B., Bullwinkle T. J., Wolfe B. S., Gilreath M. S., Forsyth C. J., Navarre W. W., and Ibba M. (2011) The tRNA synthetase paralog PoxA modifies elongation factor-P with (*R*)-β-lysine. Nat. Chem. Biol. 7, 667–669 21841797 10.1038/nchembio.632 PMC3177975

[8] Gutierrez E., Shin B. S., Woolstenhulme C. J., Kim J. R., Saini P., Buskirk A. R., and Dever T. E. (2013) eIF5A promotes translation of polyproline motifs. Mol. Cell 51, 35–45 23727016 10.1016/j.molcel.2013.04.021 PMC3744875

[9] Ude S., Lassak J., Starosta A. L., Kraxenberger T., Wilson D. N., and Jung K. (2013) Translation elongation factor EF-P alleviates ribosome stalling at polyproline stretches. Science 339, 82–85 23239623 10.1126/science.1228985

[10] Doerfel L. K., Wohlgemuth I., Kothe C., Peske F., Urlaub H., and Rodnina M. V. (2013) EF-P is essential for rapid synthesis of proteins containing consecutive proline residues. Science 339, 85–88 23239624 10.1126/science.1229017

[11] Nakada Y., and Itoh Y. (2003) Identification of the putrescine biosynthetic genes in *Pseudomonas aeruginosa* and characterization of agmatine deiminase and *N*-carbamoylputrescine amidohydrolase of the arginine decarboxylase pathway. Microbiology 149, 707–714 12634339 10.1099/mic.0.26009-0

[12] Hanfrey C. C., Pearson B. M., Hazeldine S., Lee J., Gaskin D. J., Woster P. M., Phillips M. A., and Michael A. J. (2011) Alternative spermidine biosynthetic route is critical for growth of *Campylobacter jejuni* and is the dominant polyamine pathway in human gut microbiota. J. Biol. Chem. 286, 43301–43312 22025614 10.1074/jbc.M111.307835 PMC3234850

[13] Kim S. H., Wang Y., Khomutov M., Khomutov A., Fuqua C., and Michael A. J. (2016) The essential role of spermidine in growth of *Agrobacterium tumefaciens* is determined by the 1,3-diaminopropane moiety. ACS Chem. Biol. 11, 491–499 26682642 10.1021/acschembio.5b00893 PMC5061148

[14] Chattopadhyay M. K., Tabor C. W., and Tabor H. (2009) Polyamines are not required for aerobic growth of *Escherichia coli*: preparation of a strain with deletions in all of the genes for polyamine biosynthesis. J. Bacteriol. 191, 5549–5552 19542271 10.1128/JB.00381-09 PMC2725612

[15] Green R., Hanfrey C. C., Elliott K. A., McCloskey D. E., Wang X., Kanugula S., Pegg A. E., and Michael A. J. (2011) Independent evolutionary origins of functional polyamine biosynthetic enzyme fusions catalysing *de novo* diamine to triamine formation. Mol. Microbiol. 81, 1109–1124 21762220 10.1111/j.1365-2958.2011.07757.x PMC3196669

[16] Patel C. N., Wortham B. W., Lines J. L., Fetherston J. D., Perry R. D., and Oliveira M. A. (2006) Polyamines are essential for the formation of plague biofilm. J. Bacteriol. 188, 2355–2363 16547021 10.1128/JB.188.7.2355-2363.2006 PMC1428407

[17] Burrell M., Hanfrey C. C., Murray E. J., Stanley-Wall N. R., and Michael A. J. (2010) Evolution and multiplicity of arginine decarboxylases in polyamine biosynthesis and essential role in *Bacillus subtilis* biofilm formation. J. Biol. Chem. 285, 39224–39238 20876533 10.1074/jbc.M110.163154 PMC2998088

[18] Hobley L., Kim S. H., Maezato Y., Wyllie S., Fairlamb A. H., Stanley-Wall N. R., and Michael A. J. (2014) Norspermidine is not a self-produced trigger for biofilm disassembly. Cell 156, 844–854 24529384 10.1016/j.cell.2014.01.012 PMC3969229

[19] Potter A. J., and Paton J. C. (2014) Spermidine biosynthesis and transport modulate pneumococcal autolysis. J. Bacteriol. 196, 3556–3561 25092031 10.1128/JB.01981-14 PMC4187697

[20] Flemming H. C., and Wingender J. (2010) The biofilm matrix. Nat. Rev. Microbiol. 8, 623–633 20676145 10.1038/nrmicro2415

[21] Vlamakis H., Chai Y., Beauregard P., Losick R., and Kolter R. (2013) Sticking together: building a biofilm the *Bacillus subtilis* way. Nat. Rev. Microbiol. 11, 157–168 23353768 10.1038/nrmicro2960 PMC3936787

[22] Cairns L. S., Hobley L., and Stanley-Wall N. R. (2014) Biofilm formation by *Bacillus subtilis*: new insights into regulatory strategies and assembly mechanisms. Mol. Microbiol. 93, 587–598 24988880 10.1111/mmi.12697 PMC4238804

[23] Flemming H. C., Wingender J., Szewzyk U., Steinberg P., Rice S. A., and Kjelleberg S. (2016) Biofilms: an emergent form of bacterial life. Nat. Rev. Microbiol. 14, 563–575 27510863 10.1038/nrmicro.2016.94

[24] Karatan E., Duncan T. R., and Watnick P. I. (2005) NspS, a predicted polyamine sensor, mediates activation of *Vibrio cholerae* biofilm formation by norspermidine. J. Bacteriol. 187, 7434–7443 16237027 10.1128/JB.187.21.7434-7443.2005 PMC1273002

[25] Lee J., Sperandio V., Frantz D. E., Longgood J., Camilli A., Phillips M. A., and Michael A. J. (2009) An alternative polyamine biosynthetic pathway is widespread in bacteria and essential for biofilm formation in *Vibrio cholerae*. J. Biol. Chem. 284, 9899–9907 19196710 10.1074/jbc.M900110200 PMC2665113

[26] Wortham B. W., Oliveira M. A., Fetherston J. D., and Perry R. D. (2010) Polyamines are required for the expression of key Hms proteins important for *Yersinia pestis* biofilm formation. Environ. Microbiol. 12, 2034–2047 20406298 10.1111/j.1462-2920.2010.02219.x PMC3039482

[27] Ding Y., Peng N., Du Y., Ji L., and Cao B. (2014) Disruption of putrescine biosynthesis in *Shewanella oneidensis* enhances biofilm cohesiveness and performance in Cr(VI) immobilization. Appl. Environ. Microbiol. 80, 1498–1506 24362428 10.1128/AEM.03461-13 PMC3911039

[28] Wang Y., Kim S. H., Natarajan R., Heindl J. E., Bruger E. L., Waters C. M., Michael A. J., and Fuqua C. (2016) Spermidine inversely influences surface interactions and planktonic growth in *Agrobacterium tumefaciens*. J. Bacteriol. 198, 2682–2691 27402627 10.1128/JB.00265-16 PMC5019071

[29] Böttcher T., Kolodkin-Gal I., Kolter R., Losick R., and Clardy J. (2013) Synthesis and activity of biomimetic biofilm disruptors. J. Am. Chem. Soc. 135, 2927–2930 23406351 10.1021/ja3120955 PMC3585461

[30] Branda S. S., Chu F., Kearns D. B., Losick R., and Kolter R. (2006) A major protein component of the *Bacillus subtilis* biofilm matrix. Mol. Microbiol. 59, 1229–1238 16430696 10.1111/j.1365-2958.2005.05020.x

[31] Branda S. S., González-Pastor J. E., Ben-Yehuda S., Losick R., and Kolter R. (2001) Fruiting body formation by *Bacillus subtilis*. Proc. Natl. Acad. Sci. U.S.A. 98, 11621–11626 11572999 10.1073/pnas.191384198 PMC58779

[32] Ostrowski A., Mehert A., Prescott A., Kiley T. B., and Stanley-Wall N. R. (2011) YuaB functions synergistically with the exopolysaccharide and TasA amyloid fibers to allow biofilm formation by *Bacillus subtilis*. J. Bacteriol. 193, 4821–4831 21742882 10.1128/JB.00223-11 PMC3165672

[33] Kobayashi K., and Iwano M. (2012) BslA(YuaB) forms a hydrophobic layer on the surface of *Bacillus subtilis* biofilms. Mol. Microbiol. 85, 51–66 22571672 10.1111/j.1365-2958.2012.08094.x

[34] Hobley L., Ostrowski A., Rao F. V., Bromley K. M., Porter M., Prescott A. R., MacPhee C. E., van Aalten D. M., and Stanley-Wall N. R. (2013) BslA is a self-assembling bacterial hydrophobin that coats the *Bacillus subtilis* biofilm. Proc. Natl. Acad. Sci. U.S.A. 110, 13600–13605 23904481 10.1073/pnas.1306390110 PMC3746881

[35] Hamon M. A., and Lazazzera B. A. (2001) The sporulation transcription factor Spo0A is required for biofilm development in *Bacillus subtilis*. Mol. Microbiol. 42, 1199–1209 11886552 10.1046/j.1365-2958.2001.02709.x

[36] Sekowska A., Bertin P., and Danchin A. (1998) Characterization of polyamine synthesis pathway in *Bacillus subtilis* 168. Mol. Microbiol. 29, 851–858 9723923 10.1046/j.1365-2958.1998.00979.x

[37] Sekowska A., Coppée J. Y., Le Caer J. P., Martin-Verstraete I., and Danchin A. (2000) *S*-Adenosylmethionine decarboxylase of *Bacillus subtilis* is closely related to archaebacterial counterparts. Mol. Microbiol. 36, 1135–1147 10844697 10.1046/j.1365-2958.2000.01930.x

[38] Hofer U. (2014) Biofilm diassembly revisited. Nat. Rev. Microbiol. 12, 234 10.1038/nrmicro3233 24531616

[39] Yoshida K., Yamaguchi H., Kinehara M., Ohki Y. H., Nakaura Y., and Fujita Y. (2003) Identification of additional TnrA-regulated genes of *Bacillus subtilis* associated with a TnrA box. Mol. Microbiol. 49, 157–165 12823818 10.1046/j.1365-2958.2003.03567.x

[40] Schultz A. C., Nygaard P., and Saxild H. H. (2001) Functional analysis of 14 genes that constitute the purine catabolic pathway in *Bacillus subtilis* and evidence for a novel regulon controlled by the PucR transcription activator. J. Bacteriol. 183, 3293–3302 11344136 10.1128/JB.183.11.3293-3302.2001 PMC99626

[41] Beier L., Nygaard P., Jarmer H., and Saxild H. H. (2002) Transcription analysis of the *Bacillus subtilis* PucR regulon and identification of a cis-acting sequence required for PucR-regulated expression of genes involved in purine catabolism. J. Bacteriol. 184, 3232–3241 12029039 10.1128/JB.184.12.3232-3241.2002 PMC135105

[42] Sekowska A., and Danchin A. (2002) The methionine salvage pathway in Bacillus subtilis. BMC Microbiol. 2, 8 12022921 10.1186/1471-2180-2-8 PMC113757

[43] Domínguez-Escobar J., Wolf D., Fritz G., Höfler C., Wedlich-Söldner R., and Mascher T. (2014) Subcellular localization, interactions and dynamics of the phage-shock protein-like Lia response in *Bacillus subtilis*. Mol. Microbiol. 92, 716–732 24666271 10.1111/mmi.12586

[44] Cybulski L. E., Albanesi D., Mansilla M. C., Altabe S., Aguilar P. S., and de Mendoza D. (2002) Mechanism of membrane fluidity optimization: isothermal control of the *Bacillus subtilis* acyl-lipid desaturase. Mol. Microbiol. 45, 1379–1388 12207704 10.1046/j.1365-2958.2002.03103.x

[45] Kearns D. B., and Losick R. (2005) Cell population heterogeneity during growth of *Bacillus subtilis*. Genes Dev. 19, 3083–3094 16357223 10.1101/gad.1373905 PMC1315410

[46] Chai Y., Chu F., Kolter R., and Losick R. (2008) Bistability and biofilm formation in *Bacillus subtilis*. Mol. Microbiol. 67, 254–263 18047568 10.1111/j.1365-2958.2007.06040.x PMC2430929

[47] Chai Y., Norman T., Kolter R., and Losick R. (2010) An epigenetic switch governing daughter cell separation in *Bacillus subtilis*. Genes Dev. 24, 754–765 20351052 10.1101/gad.1915010 PMC2854391

[48] Oves-Costales D., Song L., and Challis G. L. (2009) Enantioselective desymmetrisation of citric acid catalysed by the substrate-tolerant petrobactin biosynthetic enzyme AsbA. Chem. Commun. 1389–1391 10.1039/b823147h 19259597

[49] Hagan A. K., Carlson P. E. Jr., and Hanna P. C. (2016) Flying under the radar: the non-canonical biochemistry and molecular biology of petrobactin from *Bacillus anthracis*. Mol. Microbiol. 102, 196–206 27425635 10.1111/mmi.13465

[50] Woolridge D. P., Martinez J. D., Stringer D. E., and Gerner E. W. (1999) Characterization of a novel spermidine/spermine acetyltransferase, BltD, from *Bacillus subtilis*. Biochem. J. 340, 753–758 10359661 PMC1220308

[51] Park M. H., and Wolff E. C. (1988) Cell-free synthesis of deoxyhypusine: separation of protein substrate and enzyme and identification of 1,3-diaminopropane as a product of spermidine cleavage. J. Biol. Chem. 263, 15264–15269 3139668

[52] Park J. H., Wolff E. C., Folk J. E., and Park M. H. (2003) Reversal of the deoxyhypusine synthesis reaction: generation of spermidine or homospermidine from deoxyhypusine by deoxyhypusine synthase. J. Biol. Chem. 278, 32683–32691 12788913 10.1074/jbc.M304247200

[53] Weber M. H., Klein W., Müller L., Niess U. M., and Marahiel M. A. (2001) Role of the *Bacillus subtilis* fatty acid desaturase in membrane adaptation during cold shock. Mol. Microbiol. 39, 1321–1329 11251847 10.1111/j.1365-2958.2001.02322.x

[54] Winkelman J. T., Bree A. C., Bate A. R., Eichenberger P., Gourse R. L., and Kearns D. B. (2013) RemA is a DNA-binding protein that activates biofilm matrix gene expression in *Bacillus subtilis*. Mol. Microbiol. 88, 984–997 23646920 10.1111/mmi.12235 PMC3732408

[55] Diethmaier C., Pietack N., Gunka K., Wrede C., Lehnik-Habrink M., Herzberg C., Hübner S., and Stülke J. (2011) A novel factor controlling bistability in *Bacillus subtilis*: the YmdB protein affects flagellin expression and biofilm formation. J. Bacteriol. 193, 5997–6007 21856853 10.1128/JB.05360-11 PMC3194898

[56] Diethmaier C., Newman J. A., Kovács A. T., Kaever V., Herzberg C., Rodrigues C., Boonstra M., Kuipers O. P., Lewis R. J., and Stülke J. (2014) The YmdB phosphodiesterase is a global regulator of late adaptive responses in *Bacillus subtilis*. J. Bacteriol. 196, 265–275 24163345 10.1128/JB.00826-13 PMC3911264

[57] Meeske A. J., Rodrigues C. D., Brady J., Lim H. C., Bernhardt T. G., and Rudner D. Z. (2016) High-throughput genetic screens identify a large and diverse collection of new sporulation genes in *Bacillus subtilis*. PLos Biol. 14, e1002341 26735940 10.1371/journal.pbio.1002341 PMC4703394

[58] Ishii I., Takada H., Terao K., Kakegawa T., Igarashi K., and Hirose S. (1994) Decrease in spermidine content during logarithmic phase of cell growth delays spore formation of *Bacillus subtilis*. Cell. Mol. Biol. 40, 925–931 7849560

[59] Honjo M., Nakayama A., Fukazawa K., Kawamura K., Ando K., Hori M., and Furutani Y. (1990) A novel *Bacillus subtilis* gene involved in negative control of sporulation and degradative-enzyme production. J. Bacteriol. 172, 1783–1790 2108124 10.1128/jb.172.4.1783-1790.1990 PMC208669

[60] Ferrari F. A., Trach K., LeCoq D., Spence J., Ferrari E., and Hoch J. A. (1985) Characterization of the spo0A locus and its deduced product. Proc. Natl. Acad. Sci. U.S.A. 82, 2647–2651 3157992 10.1073/pnas.82.9.2647 PMC397621

[61] Trowsdale J., Chen S. M., and Hoch J. A. (1978) Evidence that spo0A mutations are recessive in spo0A−/spo0A+ merodiploid strains of *Bacillus subtilis*. J. Bacteriol. 135, 99–113 97278 10.1128/jb.135.1.99-113.1978 PMC366596

[62] López D., Vlamakis H., Losick R., and Kolter R. (2009) Cannibalism enhances biofilm development in *Bacillus subtilis*. Mol. Microbiol. 74, 609–618 19775247 10.1111/j.1365-2958.2009.06882.x PMC2983100

[63] Verhamme D. T., Murray E. J., and Stanley-Wall N. R. (2009) DegU and Spo0A jointly control transcription of two loci required for complex colony development by *Bacillus subtilis*. J. Bacteriol. 191, 100–108 18978066 10.1128/JB.01236-08 PMC2612447

[64] Li B., Lowe-Power T., Kurihara S., Gonzales S., Naidoo J., MacMillan J. B., Allen C., and Michael A. J. (2016) Functional identification of putrescine *C*- and *N*-hydroxylases. ACS Chem. Biol. 11, 2782–2789 27541336 10.1021/acschembio.6b00629

[65] Murray E. J., Strauch M. A., and Stanley-Wall N. R. (2009) sX is involved in controlling *Bacillus subtilis* biofilm architecture through the AbrB homologue Abh. J. Bacteriol. 191, 6822–6832 19767430 10.1128/JB.00618-09 PMC2772499

[66] Feichtinger K., Zapf C., Sings H. L., and Goodman M. (1998) Diprotected triflylguanidines: a new class of guanidylation reagents. J. Org. Chem. 63, 3804–3805

[67] Perego M., Spiegelman G. B., and Hoch J. A. (1988) Structure of the gene for the transition state regulator, *abrB*:regulator synthesis is controlled by the *spo0A* sporulation gene in *Bacillus subtilis*. Mol. Microbiol. 2, 689–699 3145384 10.1111/j.1365-2958.1988.tb00079.x

