# Supplementary material for: Spermidine promotes Bacillus subtilis biofilm formation by activating expression of the matrix regulator slrR
Source: J Biol Chem. 2017 May 25;292(29):12041–53. doi: 10.1074/jbc.M117.789644 (PMC5519356; doi:10.1074/jbc.M117.789644)
Supplement: Supplemental Data [file supp_292_29_12041__index.html]

Spermidine promotes Bacillus subtilis biofilm formation by activating expression of the matrix regulator slrR — Spermidine promotes Bacillus subtilis biofilm formation by activating expression of the matrix regulator slrR — Spermidine-dependent biofilm formation — Supplemental Data 

# Spermidine promotes *Bacillus subtilis* biofilm formation by activating expression of the matrix regulator *slrR*

## Supplemental Data

- Supplemental Figure S1 (.pdf, 3.2 MB) - Supplemental Figure S1
- Table S1 (.txt, 225 KB)
- Table S2 (.txt, 158 KB) - Table S2
