# Supplementary material for: Spermidine promotes Bacillus subtilis biofilm formation by activating expression of the matrix regulator slrR
Source: J Biol Chem. 2017 May 25;292(29):12041–53. doi: 10.1074/jbc.M117.789644 (PMC5519356; doi:10.1074/jbc.M117.789644)
Supplement: Supplemental Data [file 10.1074_M117.789644_jbc.M117.789644-1.pdf]

SUPPORTING INFORMATION

Spermidine promotes *Bacillus subtilis* biofilm formation by activating expression of matrix regulator *slrR*

Laura Hobley[1,2,5], Jennifer L. Wood[2], Bin Li[1,3], Sok Ho Kim[1,6], Jacinth Naidoo[3], Ana-Sofia Ferreira[2], Maxim Khomutov[4], Alexey Khomutov[4], Nicola R. Stanley-Wall[2] and Anthony J. Michael[1,3]

[1]Dept. of Pharmacology, University of Texas Southwestern Medical Center at Dallas, Texas 75390, USA; [2]Division of Molecular Microbiology, College of Life Sciences, University of Dundee, DD15EH, UK; [3]Dept. of Biochemistry, University of Texas Southwestern Medical Center at Dallas, Texas 75390, USA; [4]Engelhardt Institute of Molecular Biology, Russian Academy of Sciences, Vavilov St. 32, Moscow 119991, Russia; [5]Current Address: School of Medicine, Dentistry and Biomedical Sciences, Queen's University, Belfast, UK; [6]Current Address: Realtox Labs, Reisterstown, MD 21136, USA

Tables S1 & S2

Microarray data for sample set 1,2,3 (Table S1) and 4,5,6 (Table S2) comparing Δ*speD* (Δ*S*-adenosylmethionine decarboxylase) cells with wild-type (parental) *B. subtilis* NCIB3610 cells.

RNA was harvested from mid-exponential phase cells. Biological replicates performed in parallel (samples speD-1,2,3 and 3610-1,2,3 – Table S1). The experiments were then repeated from freshly inoculated cultures one week later (samples speD-4,5,6 and 3610-1,2,3 – Table S2). Genes encoding mRNAs that were changed in steady-state abundance more than 1.5-fold between the wildtype NCIB3610 and Δ*speD* strains are listed. Strains were grown at 37°C in polyamine-free chemically-defined liquid growth medium (MSgg).

Figure S1

NMR spectra (^{1}H and ^{13}C) for the dihydrobromide salt of noragmatine (Gua-(CH2)3-NH2) and ditosylate salt of homoagmatine (Gua-(Ch2)5-NH2).

Figure S1

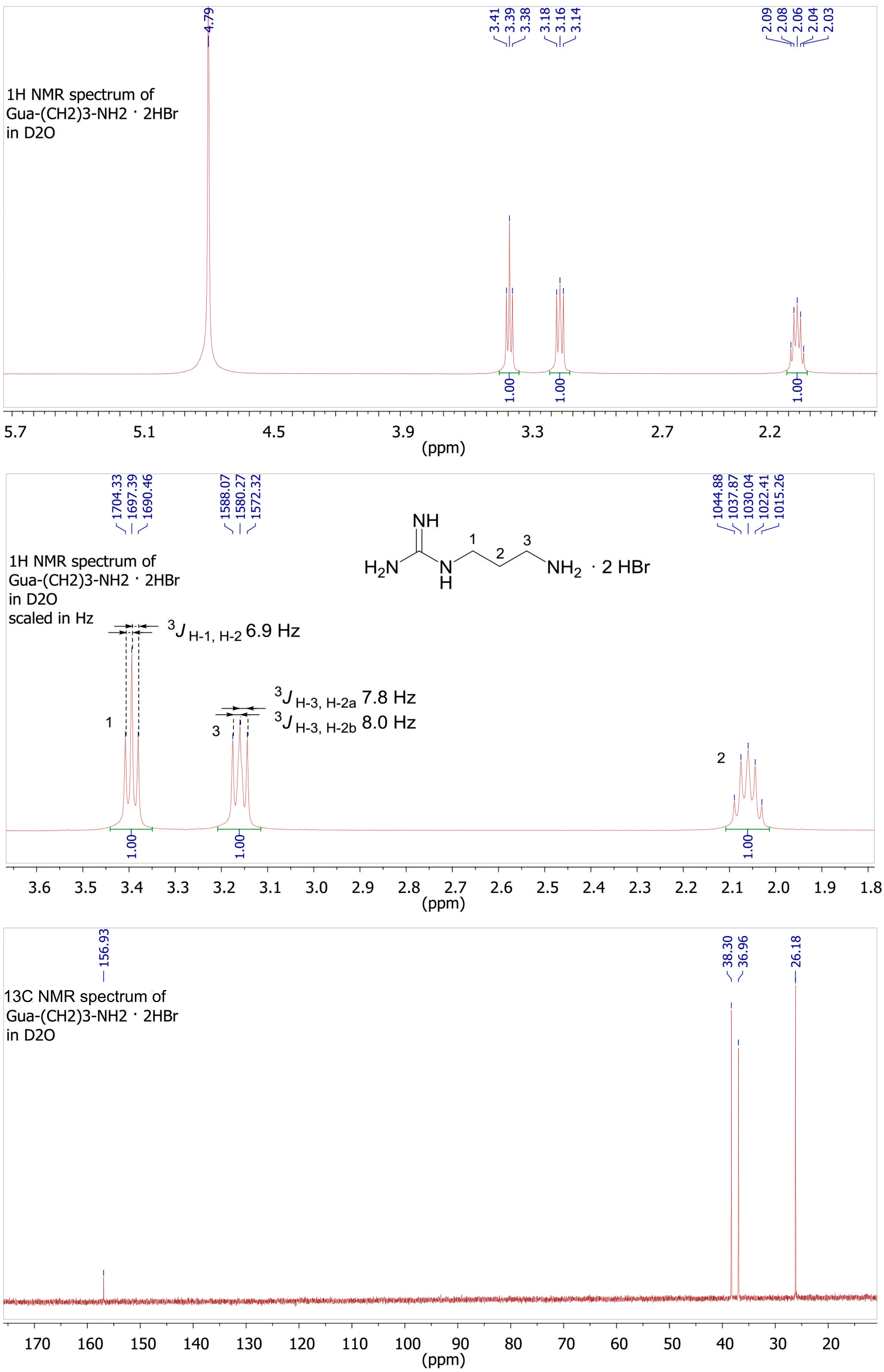
1H NMR spectrum of
Gua-(CH2)3-NH2 · 2HBr
in D2O
4.79
3.41
3.39
3.38
3.18
3.16
3.14
2.09
2.08
2.06
2.04
2.03
1.00
1.00
1.00
5.7
5.1
4.5
3.9
3.3
2.7
2.2
(ppm)
1H NMR spectrum of
Gua-(CH2)3-NH2 · 2HBr
in D2O
scaled in Hz
1704.33
1697.39
1690.46
1588.07
1580.27
1572.32
1044.88
1037.87
1030.04
1022.41
1015.26
NH
H_2N
N
H
1
2
3
NH_2 · 2 HBr
$^3J_{H-1, H-2}$ 6.9 Hz
$^3J_{H-3, H-2a}$ 7.8 Hz
$^3J_{H-3, H-2b}$ 8.0 Hz
1
3
2
1.00
1.00
1.00
3.6
3.5
3.4
3.3
3.2
3.1
3.0
2.9
2.8
2.7
2.6
2.5
2.4
2.3
2.2
2.1
2.0
1.9
1.8
(ppm)
13C NMR spectrum of
Gua-(CH2)3-NH2 · 2HBr
in D2O
156.93
38.30
36.96
26.18
170
160
150
140
130
120
110
100
90
80
70
60
50
40
30
20
(ppm)

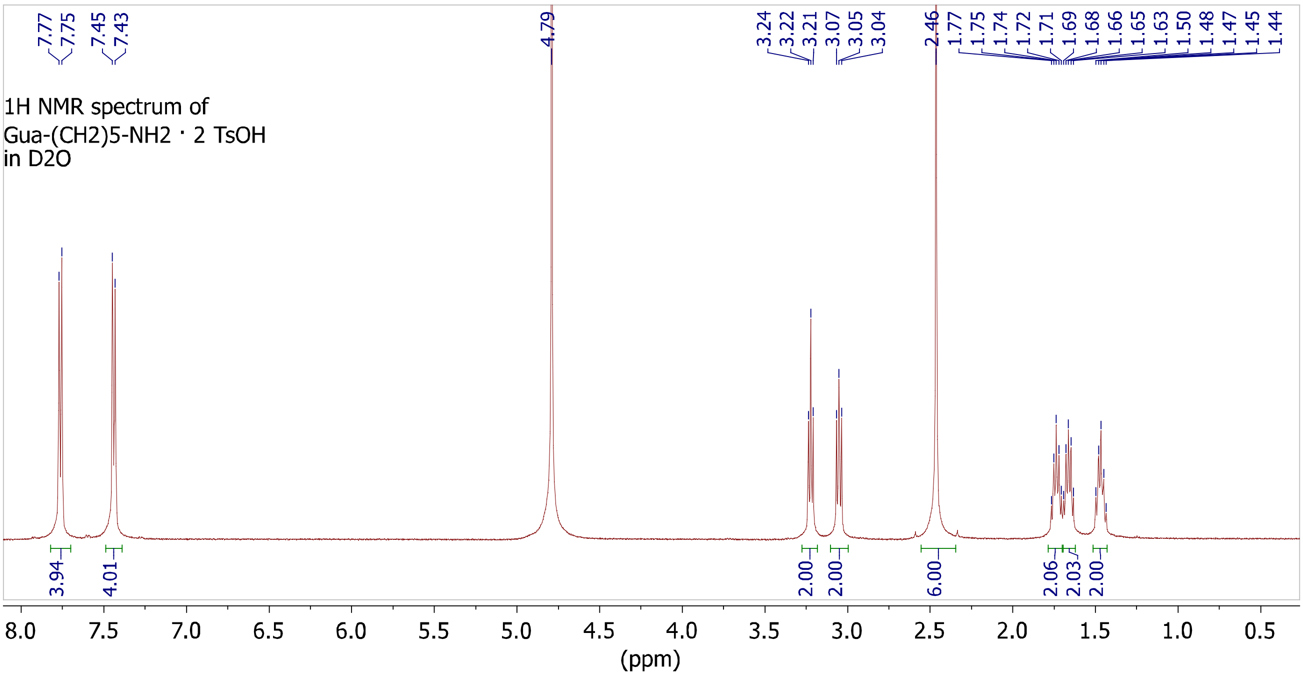
1H NMR spectrum of
Gua-(CH2)5-NH2 · 2 TsOH
in D2O
7.77 7.75 7.45 7.43 4.79 3.24 3.22 3.21 3.07 3.05 3.04 2.46 1.77 1.75 1.74 1.72 1.71 1.69 1.68 1.66 1.65 1.63 1.50 1.48 1.47 1.45 1.44
3.94 4.01 2.00 2.00 6.00 2.06 2.03 2.00
(ppm)

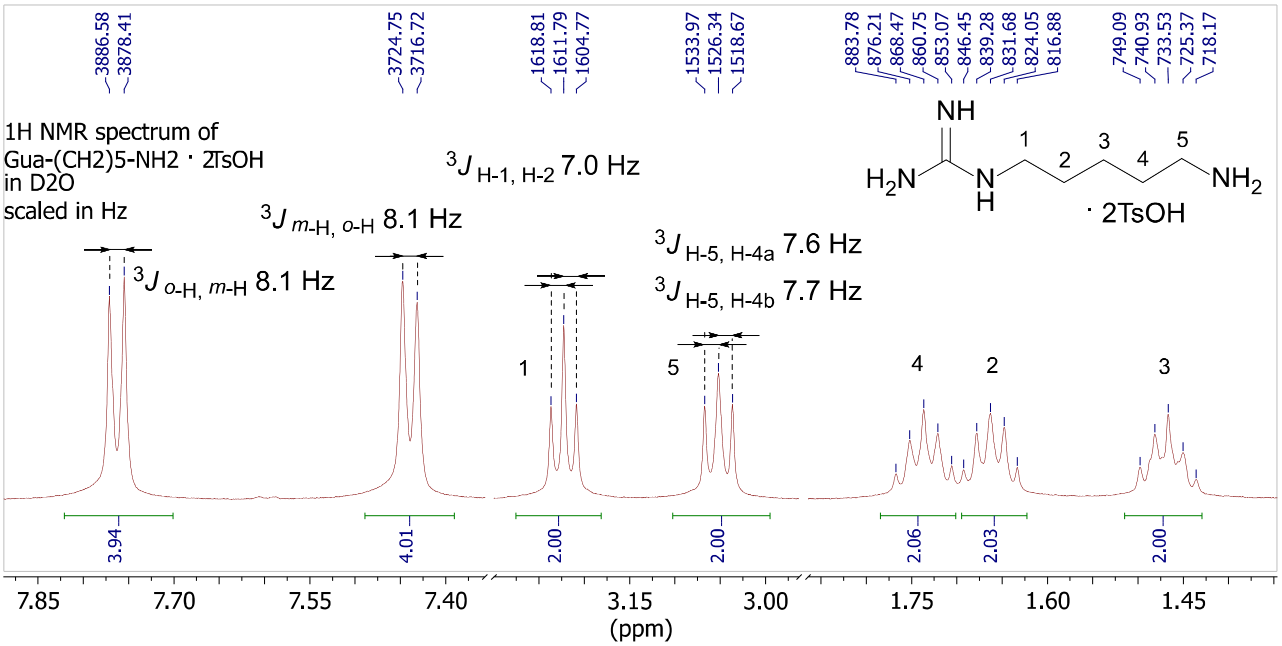
1H NMR spectrum of
Gua-(CH2)5-NH2 · 2TsOH
in D2O
scaled in Hz
3886.58 3878.41 3724.75 3716.72 1618.81 1611.79 1604.77 1533.97 1526.34 1518.67 883.78 876.21 868.47 860.75 853.07 846.45 839.28 831.68 824.05 816.88 749.09 740.93 733.53 725.37 718.17
$^3J_{o\text{-H}, m\text{-H}}$ 8.1 Hz
$^3J_{m\text{-H}, o\text{-H}}$ 8.1 Hz
$^3J_{\text{H-1, H-2}}$ 7.0 Hz
$^3J_{\text{H-5, H-4a}}$ 7.6 Hz
$^3J_{\text{H-5, H-4b}}$ 7.7 Hz
NH
H_2N
N
H
NH_2
· 2TsOH
1 2 3 4 5
3.94 4.01 2.00 2.00 2.06 2.03 2.00
(ppm)

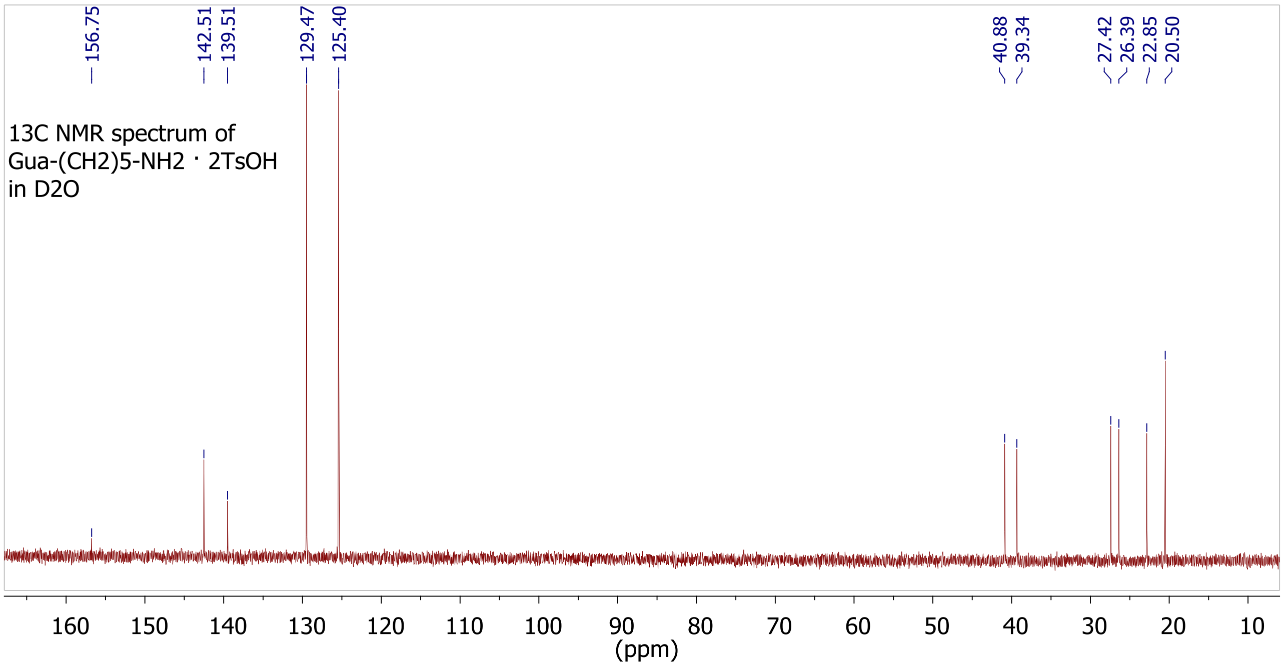
13C NMR spectrum of
Gua-(CH2)5-NH2 · 2TsOH
in D2O
156.75 142.51 139.51 129.47 125.40 40.88 39.34 27.42 26.39 22.85 20.50
(ppm)
